# Gender-affirming orchiectomy in transgender and non-binary individuals: A large cohort study with middle- to long-term follow-up

**DOI:** 10.1080/26895269.2024.2396939

**Published:** 2024-09-10

**Authors:** Wouter B. van der Sluis, Jan Maerten Smit, Tim Schäfer, Mark-Bram Bouman

**Affiliations:** a Department of Plastic, Reconstructive and Hand Surgery, Amsterdam University Medical Center, Amsterdam, the Netherlands; b Center of Expertise on Gender Dysphoria, Amsterdam University Medical Center, location VUmc, Amsterdam, the Netherlands; c Department of Plastic Surgery, Gender Clinic, Bosch en Duin, The Netherlands; d Department of Plastic Surgery, University Medical Centre Groningen, University of Groningen, Groningen, Netherlands

**Keywords:** Gender dysphoria, gGAS, non-binary, orchiectomy, transgender

## Abstract

**Introduction:**

Some transgender individuals opt for gender-affirming orchiectomy. Data on postoperative outcomes in the transgender population are scarce.

**Methods:**

All individuals who underwent gender-affirming orchiectomy between 01-2010 and 01-2024 were retrospectively identified at two centers that provide surgical transgender care. Individual demographics, motivations, intra- and postoperative complications, reoperations and postoperative regret were recorded. Surgical risk factors were identified using logistic regression analysis.

**Results:**

A total of 119 individuals were retrospectively identified with a median clinical follow-up of 2.3 years (range 0.3–9.4). A total of 109 identified as transgender woman, eight as non-binary and two as agender. 18 (15%) individuals opted for sperm cryopreservation before surgery. The postoperative course was without complications in 102 (86%) individuals. Complications comprised hemorrhage (*n* = 5, 4%, Clavien-Dindo 3b) and abscess formation (*n* = 7, 6%, Clavien-Dindo 2), (*n* = 1), 3a (*n* = 3) and 3b (*n* = 3))). In the follow-up time, seven individuals underwent vaginoplasty, two vulvoplasty and two were on the waiting list for vaginoplasty. There were no cases of regret. A BMI >35 was identified as risk factor for infectious complications (*p* = .045, OR 9.8, 95% CI 1.0–99.3).

**Discussion:**

Gender-affirming orchiectomy is a simple and safe procedure. It can be performed as standalone gender-affirming surgical procedure, or as bridge to another gender-affirming procedure.

## Introduction

Transgender individuals assigned male at birth may opt for genital gender-affirming surgery. This may comprise bilateral orchiectomy, vulvoplasty or vaginoplasty (Van der Sluis et al., 2021). Bilateral orchiectomy is the least invasive surgical option. During surgery, both testicles are removed. After surgery, individuals do not need to continue antiandrogens (testosterone blockers) and are infertile. In the Standards of Care, five criteria are formulated for eligibility for orchiectomy in transgender individuals (Coleman et al., 2022):

1. There has to be persistent, well-documented gender dysphoria,
2. An individual has to be capable to make a fully informed decision and consent for surgery,
3. An individual has to have the age of majority in a given country
4. If there are significant (mental) health concerns, they must be well-controlled
5. An individual has at least 12 continuous months of hormone therapy and adequate testosterone blocking. This introduces a period of reversible testosterone suppression, before the orchiectomy procedure.

As orchiectomy is an irreversible procedure, we recommend preoperative fertility counseling.

Though frequently performed, little is known on (longer-term) outcomes of this procedure. Large cohort studies on this procedure, motivations, outcomes and reoperations, are lacking in current literature.

## Methods

### Surgical procedure

The surgical procedure, well-described elsewhere, is performed under general anesthesia or spinal anesthesia (Francis et al., 2020). After sterile draping, a midline scrotal incision is made. This incision style is chosen not to hinder a future vaginoplasty procedure. The testicles are freed from surrounding tissue, and dissection is performed to free the spermatic cord to the external inguinal ring. There, it is ligated, and cut. The remaining stump is infiltrated with local anesthesia, where the genital branch of the genitofemoral nerve is located. Hemostasis is performed and the wound is closed in layers. Generally, the procedure can be performed in daycare.

### Chart review

Al individuals that underwent gender-affirming orchiectomy between 01-2010 and 01-2024 were retrospectively identified at two centers that provide surgical transgender healthcare, The Amsterdam University Medical Center (Amsterdam, The Netherlands) and Gender Clinic (Bosch en Duin, The Netherlands). A retrospective chart study was conducted, recording

- Individual demographics (self-reported gender identity, age at surgery, BMI at surgery, intoxications, history of smoking, history of puberty suppression, comorbidities, history of mental health issues, sperm cryopreservation before surgery)
- motivation for gender-affirming orchiectomy
- intra- and postoperative complications, subcategorized according to the Clavien-Dindo classification of surgical complications
- reoperations and
- postoperative regret

### Statistical analysis

Normally-distributed continuous variables were presented as means with standard deviations. Non-normally-distributed continuous variables as medians with ranges. Categorical data were presented as frequencies and percentages. Risk factors with regard to bleeding and infectious complications were identified using logistic regression analysis in SPSS version 28.0.

### Ethics

This research and study protocol conforms to the ethical norms and standards as formulated in the Declaration of Helsinki, including institutional ethics committee approval (METC2014322). Included individuals provided written informed consent for use of their anonymous demographic and surgical data.

## Results

### Demographics

**Figure 1.**
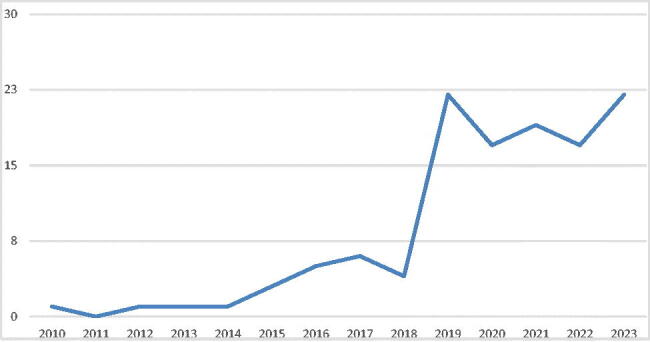
Performed gender-affirming orchiectomy procedures per year.

A total of 119 individuals were retrospectively identified that underwent gender-affirming orchiectomy in the study time period. An overview of performed procedures per year is presented in Figure 1. An overview of demographics is presented in Table 1.

**Table 1.** Participant demographics.

Total number of included individuals	119
Gender-identity at time of surgery, *n* (%)	
Transgender women	109 (92%)
Non-binary	8 (7%)
Agender	2 (2%)
Median age (range)	28 (18–69)
Mean BMI (kg/m^2^) ± SD	25.6 ± 6.0
BMI, categorized, *n* (%)	
18–25	66 (55%)
25–30	26 (22%)
30–35	17 (14%)
>35	10 (8%)
Intoxications, *n* (%)	
History of smoking/active smoker	40 (34%)
History of alcohol abuse	2 (2%)
History of marihuana abuse	18 (15%)
Medical history, *n* (%)^a^	
Diabetes	4 (3%)
Myocardial infarction	1 (1%)
Pulmonary embolism	2 (2%)
Hypercholesterolemia	1 (1%)
Hypertension	2 (2%)
Relevant surgical history, *n* (%)	
Inguinal hernia surgery	9 (8%)
Orchidopexy	3 (3%)
History of mental health issues, *n* (%)^a^	
Autism spectrum disorder	16 (13%)
ADHD	6 (5%)
Depression	22 (18%)
Psychosis	4 (3%)
Reason for orchiectomy, *n* (%)	
No desire for vaginoplasty because of no genital dysphoria	34 (29%)
Wants vaginoplasty, but does not meet institutional requirements	31 (26%)
BMI too high for institutional requirements (>30)	19 (16%)
Cannot stop smoking	7 (6%)
Interfering somatic or mental health issues	5 (4%)
Preceding step to (possible) future vaginoplasty	46 (39%)
Finds vaginoplasty too risky	8 (7%)
Sperm cryopreservation before surgery, *n* (%)	18 (15%)

^a^Some individuals had multiple comorbidities. SD Standard Deviation.

### Surgical outcomes

The mean surgical duration was 15 ± 4 min. The median clinical follow-up was 2.3 years (range 0.3–9.4). The postoperative course was without complications in 102 (86%) individuals. Complications comprised mainly hemorrhage (*n* = 5, 4%) and abscess formation (*n* = 7, 6%). An overview of complications and Clavien–Dindo categorization is presented in Table 2. Logistic regression analysis showed that higher BMI was a risk factor for infectious complications (*p* = .047, OR 1.13 95%CI 1.005–1.285). When taken as a categorical variable (BMI <25, 25–30, 30–35 or >35), a BMI >35 was identified as risk factor for infectious complications (*p* = .045, OR 9.8, 95% CI 1.0–99.3).

**Table 2.** Postoperative complications.

Short-term complications (<3 wk)	
Hemorrhage, for which return to operation room (C-D 3b)	5 (4.2%)
Hematoma (C-D 1)	3 (2.5%)
Abscess formation	7 (5.8%)
C-D 2	1 (0.8%)
C-D 3a (Incision and drainage under local anesthesia)	3 (2.5%)
C-D 3b (Incision and drainage under general anesthesia)	3 (2.5%)
Wound dehiscence (C-D 1)	1 (0.8%)
Seroma (C-D 1)	1 (0.8%)
Long-term complications (>3 wk)	
Chronic pain operative area >3 months after surgery (C-D 1)	2 (1.7%)
Postoperative regret	–

C-D Clavien-Dindo.

In the follow-up time, a total of 9 individuals underwent further genital gender-affirming surgery (vaginoplasty (*n* = 7) or vulvoplasty (*n* = 2)) and two were on the waiting list for vaginoplasty. Of these nine individuals, four chose orchiectomy as bridge to vaginoplasty, three had a too high BMI initially for a vaginoplasty procedure, one was a smoker (which is regarded a contraindication for vaginoplasty in our center), one found a vaginoplasty procedure too risky initially. There were no cases of regret.

## Discussion

In this article, a total of 119 individuals were described that underwent gender-affirming orchiectomy. A total of 102 (86%) individuals had an uneventful postoperative course. Complications comprised mainly hemorrhage (*n* = 5, 4%) and abscess formation (*n* = 7, 6%). These surgical outcomes are very much in line with available literature on (short-term) postoperative complications (Table 3). Current literature on surgical outcomes after gender-affirming orchiectomy is very scarce. It comprises of a few retrospective cohort studies, one of them by our own research group, with short-term follow-up and a few retrospective database studies with overlapping study time periods (Hana et al., 2020; Russell et al., 2023; Saltman et al., 2023; Van der Sluis et al., 2020). All are of a retrospective nature and have a short-term follow-up period.

**Table 3.** Current literature on gender-affirming orchiectomy complications.

Author	Population	Study type	Outcomes	Follow-up
Van der Sluis et al. (2020)	*N* = 43	Retrospective cohort	*N* = 39 (91%) No complications *N* = 3 (7%) Scrotal abcess *N* = 1 (2%) Surgical site infection	Median 7.6 m (range 0.4–77.6)
Hana et al. (2020)	*N* = 16	Retrospective cohort	*N* = 15 (94%) No complications *N* = 1 (6%) Sperm granuloma	30 d
Russell et al. (2023)	*N* = 241	Retrospective database	*N* = 3 (1%) organ surgical site infection *N* = 2 (1%) sepsis *N* = 3 (1%) superficial surgical site infection	30 d
Saltman et al. (2023)	*N* = 246	Retrospective database	*N* = 3 (1%) organ surgical site infection *N* = 2 (1%) sepsis *N* = 2 (1%) superficial surgical site infection *N* = 5 (2%) Return to the operation room *N* = 7 (3%) unplanned readmission	30 d

d: days, m: months.

Since 2018 a steep increase of performed gender-affirming orchiectomy procedures was observed. This is can be due to the general increase of gGAS requests, a more non-binary view of gGAS and/or a more accepted role of orchiectomy as standalone gGAS procedure. Individual motivations for gender-affirming orchiectomy vary. In this study, 29% of individuals regarded orchiectomy as a standalone gender-affirming procedure and did not wish to undergo vulvoplasty or vaginoplasty because of lack of genital dysphoria. A total of 39% of individuals regarded orchiectomy as a preceding step to (possible) future vaginoplasty. Within the study time period, four of them actually underwent a vaginoplasty procedure. This group should be informed that when they have a vaginoplasty procedure, there is a higher risk of needing additional skin grafts (Sineath et al., 2022).

Obesity and transgender surgery is a controversial topic. Many surgical centers have institutional requirements with regard to BMI for specific procedures, for instance vaginoplasty. However, these requirements are frequently not based on empirical evidence (Brownstone et al., 2021; Linsenmeyer & Garwood, 2023). In this study, a total of 16% of individuals that underwent gender-affirming orchiectomy actually desired a vaginoplasty or vulvoplasty procedure but did not meet institutional BMI requirements for this. This means that these requirements affect the surgical path of the individual and may be seen as a barrier for surgical care. However, in this study, a BMI >35 was identified as risk factor for infectious complications after gender-affirming orchiectomy. Therefore, obese individuals should be well-informed about possible risks of surgery.

Strengths of this study are the relatively large number of included individuals, the completeness of data and the middle- to long-term follow-up time. Weaknesses of this study comprise the retrospective nature and the lack of self-reported outcomes. The latter is subject of future research.

To conclude, gender-affirming orchiectomy is a simple and safe procedure. It can be performed as standalone gender-affirming surgical procedure, or as bridge to another gender-affirming procedure.
